# Medico-Clinical Notes

**Published:** 1840-07

**Authors:** 


### ART. VIII.

1. *Adversaria Medico-Clinica. Edidit* F. G. LIPPICH, M.D. *Series prima.* Fascic. I., *Morbi Lubeanorum Anno* 1828, *observati.—Patavii,* 1836. 8vo, pp. 144. *Series secunda.* Fascic. I., *Annales Scholæ Medico-Clinicæ Patavinæ, Annus* 1834-5.—*Patavii,* 1837. 8vo, pp. 182.

*Medico-Clinical Notes.* By F. G. LIPPICH, M.D. *First Series.* Fascic. I., *Diseases observed at Laybach, during the Year* 1828. *Second Series.* Fascic. I., *Annals of the Medico-Clinical School of Padua, during the Year* 1834-5. *Padua,* 1836-7.

AFTER a residence of twelve years in Laybach, as physician in ordinary, Signor Lippich had determined to publish the results of his experience, when he was appointed to the clinical chair in the university of Padua; which appointment, by the increased facilities for medical observation that it has afforded him, has induced him to change his original plan, and to bring out together an account of one year of his popular practice in Laybach, and of one year's clinical practice in Padua, under the idea that a comparative view of the diseases and their treatment, in two distant and different places, would be more interesting and instructive than a history of either published separately.

The first series, accordingly, consists of a register, month by month, of the most prevalent diseases of Laybach, in the year 1828, preceded by a summary of the meteorological changes during the same time, with which, after the manner of Sydenham, Signor Lippich attempts to connect them. But after all his observations and *ex post facto* conclusions, he does not appear to have established, even to his own satisfaction, anything more important or certain, than that great variations of the thermometer induce head affections, and, when combined with those of the barometer, chest disease; that humidity disorders the abdominal viscera, and electrical changes are attended by eruptive diseases. The want of arrangement and numerical details rendering this fasciculus quite useless for statistical purposes, we shall merely extract a few particulars, that we think may be worth the notice of our readers.

Eight cases of *trismus nascentium* are given, of which five occurred in January, and one in March, May, and November; and all of them, except that in May, were traceable to cold, and exhibited, after death, marks of cerebral congestion, and even of apoplexy. We do not rely much upon this result of a disease, in which respiration is so much impeded by the convulsions of the respiratory muscles, for the explanation of its pathology,—but we cannot help thinking that too great stress has been laid by Drs. Clarke and Colles upon the influence of an impure atmosphere in the production of this complaint, contrary to the analogy of the same disease, in its idiopathic form in the adult, which is well known to be frequently produced by cold.

Upon the use of *tartar emetic in bronchitis and pneumonia,* Signor Lippich observes, that in the former this remedy acts by inducing nausea and vomiting, with a consequent removal of the obstructing mucus; but that tolerance is established in the latter, because in pure pneumonia it is very difficult to excite vomiting. From our own experience we should say, that when it does not act upon the bowels, it has a directly sedative action upon the heart, and, if combined with opium, a most powerful sudorific effect also.

Among the cases given at some length, is one of profuse *menorrhagia*, in a young girl, produced by the irritation of hardened fæces; another of *ascites* and extreme cachexia, from enlarged spleen, cured by the patient getting drunk with gin, or a spirit distilled from the juniper; and a third of *starvation*, of an old man of seventy-nine, whose stomach and intestines, after a fatal fast of three days, were found ulcerated and highly inflamed.

From the end of June till October, a severe and fatal epidemic *dysentery* prevailed; the most remarkable point attending which was its equal virulence 3000 feet above the level of the sea, on the exposed flanks of the mountains, and on the banks of the marshes from which it first arose. Many of the cattle in the neighbouring villages were affected at this time, with mortification of the spleen; and several persons who incautiously handled the bodies, or ate the flesh of these animals, contracted carbuncles (anthraces) that proved fatal to some of them. Many dogs went mad at this time, and bit several persons; but by speedy and renewed applications of the cautery, both actual and potential, all ill effects were prevented.

In his second series, Signor Lippich, after giving a slight medico-topographical sketch of the city of Padua, proceeds to treat separately of the various diseases presented to him during the medical year 1834-5. But although the number of the cases is given, there is the same want of a proper arrangement that we noticed in the former series, and hence there is but little that we deem worth extracting.

The city of Padua, said to have been founded 1183 years B. C., is situated in an extensive plain, at an elevation of thirty-two feet, French, above the sea, on a loamy and quickly-drying soil, intersected by various branches of the river Brenta. The country slopes from the north and west, towards the sea on the east, and the marshes between the Po and the Adige on the south, on which sides it is subject to inundations, which, however, are carried off by channels formed for the purpose. Vegetation is remarkably luxuriant, particularly in the vineyards and corn-fields. The mean height of the barometer is 28·1; of the thermometer, 56° 66′, F. The number of rainy days is 105 in the year, which yield 24° 11′ 8″, by the rain gauge. The prevailing winds, in winter, are from the north and east; in the summer, from the south. The north winds often bring rain, and cold weather; but snow is rare. The dampest weather occurs in autumn, but the greatest quantity of rain falls in October, June, and May; the least in July, August, and November. During the spring, which is very short, sudden transitions to the heat of summer frequently happen; but the summer heats rarely rise above 86°, F., though they often do not fall below 77°, F. The walls of the city are six miles in circuit, comprising 4,193 houses, with gardens, fields, and open spaces. The suburbs contain 2,527 houses. The population of the whole municipality is 55,000. The deaths are 1,550, and the births 1,590. (This gives eight inhabitants to a house; the deaths nearly one in 35·5, and the births one in 34·6, nearly.) The greatest mortality occurs in winter, particularly among children under one year, who constitute one third of the whole number of deaths, and two thirds of them die in the winter quarter.

Intemperance in wine and venereal pleasures is chiefly confined to the lowest classes; of the hospital patients, however, two thirds of the males are addicted to drinking, and very few of them have not been affected with some venereal complaint. The prevailing diseases are agues, sometimes of a malignant character, gastric remittents, rheumatism, catarrhs, occasional erysipelas, pneumonia, pleurisy, hepatitis, &c. Diseases of the heart and great vessels are pretty frequent; and there is abundance of scrofula, rickets, chlorosis, and especially of worms, though tæniæ are rare. Disorders arising from venous congestion, as hemorrhoids, and other fluxes, both bloody and mucous, particularly leucorrhœa, are very common, from whence, and from agues, obstructions of the abdominal viscera arise. (Here the effect is put for the cause.) Pellagra, endemic in Upper Italy, is here confined to the peasantry. There are no acute endemic exanthemata, nor the miliary eruption of the Veronese. Scarlatina belongs more to the Alpine provinces, and is only sporadic, in general, in these parts. Towards autumn, diarrhœa and dysentery,—in winter, hooping-cough, usually spring up.

The civil hospital is built upon an eminence outside the town, facing, on the north, an arm of the Brenta, where the noise of an adjacent mill (says Signor L.) is a great obstacle to stethoscopic examinations. There are two clinical wards, situated one over the other, that for males being the lower, containing, together, twenty-four beds.

Directing his attention to the influence of the weather upon disease, Signor Lippich gives the following account of the two periods of six months, in the scholastic year 1834-5, beginning with the autumn and winter months. At this time the weather was variable, and the sky cloudy, the wind blowing chiefly from the north and east. The mean temperature, from the beginning of November to the end of March, was 39°, F., barometer 28° 3′ 13″. The greatest difference of the thermometer was 12 degrees, of the barometer one inch and seven lines. The autumn and winter were colder, and the air was drier and heavier than usual. Under this influence the diseases, particularly the more acute, were of an inflammatory character; and an epidemic variola prevailed. At the commencement of the second half-year, the weather continued wintry, and on the 17th of April snow fell, in sufficient quantity to cover the fields. It was changeable up to the summer, which was dry until August, when the gauge indicated more rain than in the four previous months. The winds, as in the winter, blew chiefly from the north and east: mean temperature, 63° 11′; barometer, 28° 1′ 27″. The mean temperature of July was 78°, and the variations of the thermometer, in May, were equal to those of the barometer in the same month, being 63° 5′ of the former, to five inches of the latter.

The inflammatory forms of disease subsiding, now gave place to gastric disorders, which assumed a bilious character as the summer advanced. Very few vernal agues remained; but towards the close of the summer all fevers, of whatsoever kind, exhibited a great tendency to an intermittent type. The gastric disorders continued through the summer, and even up to winter, so prevalent, that few, whether febrile or not, were unattended by a vitiated state of the gastro-enteric secretions. This was remarked in almost all the dissections throughout the year, and many instances of enlargement of the iliac follicles were met with.

The connexion between abdominal congestions and attacks of ague has been noticed by most writers, but, as we remarked in our review of his work on diagnosis, (*British and Foreign Med. Rev.* Vol. VI. p. 128,) no person, we believe, previous to M. Piorry, has considered such congestions, of the spleen in particular, as the proximate cause of the febrile paroxysms. The observations, however, of Signor Lippich tend strongly to confirm this opinion. In speaking of the intermittent fevers of Laybach he says, that there was uneasiness of the left hypochondrium, in all the quotidians, in most of the quartans, and in some of the tertians; in which last, however, the right side was generally affected. Now, as the enlargement of the spleen is with difficulty distinguished in many cases of ague, it might be that it was present in such tertians when it was not sufficiently marked to induce uneasiness, or a prominent tumour, or where the coexistence of a more painful affection of the liver masked the disease of the less sensible spleen. Signor Lippich accounts for the more frequent occurrence of ague in women who have passed the menstrual period, upon the principle that abdominal congestion, produced by the cessation of the menses, causes an irritation of the ganglionic nerves, that exhibits itself in the form of ague. After his arrival in Padua, he adopted in such cases the Italian plan, of applying leeches to the anus; and he states, that the results are highly favorable to this method of relieving the abdominal viscera. At the same time, he does not neglect other means, but makes use of mercurial inunctions, and the internal administration of calomel and purgatives. Many of the cases were greatly influenced by the sudden cessation and return of the hemorrhoidal and menstrual discharges. Contrary to observation in our more northern climate, the number of continued fevers was greater in summer than in winter; and though none originated from, yet many passed into, intermittents, especially during the dog-days.

Although Dr. Lippich is so firm a believer in the doctrine of crises, that he divides diseases, in his Pathological Proem, into *phanero-critici,* and *crypto-critici morbi,* yet, in his account of continued fevers, he adduces but a few instances (eight out of twenty-nine) of the termination of the complaint by a critical discharge. Of these, two occurred with a flow of urine, and one of them with sweat also, on the twentieth and fortieth days; two by diarrhœa, on the fifth and eleventh; one began on the eleventh, by the return of the catamenia, and was completed on the seventeenth, by diarrhœa; one on the fourteenth, by piles; and one by vomiting, on the fourteenth. Even of these eight, those that occurred on the eleventh, fourteenth, seventeenth, and fortieth, were not perfect crises, as the convalescence did not immediately succeed the discharges. The other four must be admitted as real crises; and though we should be sorry to recommend the inert practice of a *médecine expectante,* yet we cannot help thinking it one better calculated to exhibit the powers of nature over febrile diseases than our *nimia diligentia,* by which, we have no doubt, many a critical effort for relief is obstructed and overpowered; the medical attendant considering it as an exasperation, or complication of the malady, and treating it accordingly. The frequency of local inflammations in the fevers of this country, the improvement in our pathological knowledge of them, and the influence of the doctrines of Broussais, have, we think, contributed to this disregard of some of their most remarkable phenomena, in modern works on fever, and among the present race of practitioners; but we would still beg to recall the attention of our brethren to the admirably exact descriptions of disease left us by the ancients, and to the consideration that such close observers of symptoms could hardly have been mistaken in a matter of such importance, that it formed the basis both of their pathology and treatment. Seven cases of delirium tremens were met with, in men between the ages of twenty-eight and forty-four. This disease has only lately been observed in Italy, having been introduced, together with the use of spirits, by the foreign armies that have occupied that politically-debased country. It assumes more of a sthenic character than in northern Europe and America, and instead of opium, is best treated by venesection, cold applications, and emetics, which Dr. Lippich attributes to the greater irritability and tendency to inflammatory disease of the Italian constitution.

Three cases of that remarkable disease, pellagra, occurred, one of which, of two months' duration, was completely cured in twenty days; and both the other patients were so much relieved, as to warrant a belief in their continuing free from the complaint, if unexposed to its exciting causes. In all, the mind was affected with a melancholy stupor and indifference, with great proneness to suicide; indeed one patient, as soon as he entered the hospital, threw himself into the river, and was drowned. The treatment consisted of tonics and aperients, with leeches to the anus, to relieve abdominal congestions, together with an improved diet. Signor Lippich concurs with the Italian physicians, in attributing this disease to severe labour on an unhealthy soil, under a burning sun, with insufficient food, and all the other evils of the lowest poverty. In its commencement, it is attended with febrile and inflammatory symptoms, but soon degenerates into a cachectic form, analogous to scurvy, and terminates in insanity, of a melancholy and stupid kind. It seems to be hereditary.

